# Soluble Epoxide Hydrolase Inhibitor: A Novel Potential Therapeutic or Prophylactic Drug for Psychiatric Disorders

**DOI:** 10.3389/fphar.2019.00420

**Published:** 2019-04-24

**Authors:** Qian Ren

**Affiliations:** ^1^ Department of Human Anatomy, Hebei Medical University, Shijiazhuang, China; ^2^ Center of Stem Cell and Immune Cell Research, Institute of Medical and Health Science, Hebei Medical University, Shijiazhuang, China

**Keywords:** depression, antidepressant, schizophrenia, soluble epoxide hydrolase, inflammation

## Abstract

Psychiatric disorders, including depression and schizophrenia, affect millions of individuals worldwide. However, the precise neurobiology of psychiatric disorders remains unclear. Accumulating evidence suggests that various inflammatory processes play a key role in depression and schizophrenia, and that anti-inflammatory drugs exert a therapeutic effect in patients with psychiatric disorders. Epoxyeicosatrienoic acids (EETs) and epoxydocosapentaenoic acids (EDPs) have potent anti-inflammatory properties. These mediators are broken down into their corresponding diols by soluble epoxide hydrolase (sEH), and inhibition of sEH enhances the anti-inflammatory effects of EETs. Therefore, sEH may play a key role in inflammation, which is involved in psychiatric disorders. Recent studies have shown that abnormal levels of sEH may be involved in the pathogenesis of certain psychiatric diseases, and that sEH inhibitors exhibit antidepressant and antipsychotic activity. The present review discusses the extensive evidence supporting sEH as a therapeutic target for psychiatric diseases, and the clinical value of sEH inhibitors as therapeutic or prophylactic drugs.

## Introduction

Depression and schizophrenia are severe and chronic debilitating psychiatric diseases that affect millions of individuals worldwide, with >300 million individuals of all ages affected by depression and nearly 800,000 dying each year from suicide (World Health Organization, 2018a). Similarly, schizophrenia affects >21 million individuals, and the probability of death in this patient population is 2 to 3 times higher than that in the general population (World Health Organization, 2018b). Although current clinical antidepressants and antipsychotics have been shown to be effective in the treatment of depression and schizophrenia, at least one-third of individuals with depression do not fully respond to medications, and antipsychotics have no beneficial effects on negative symptoms and cognitive impairments (Hashimoto, 2014; Steinert et al., 2014; Biesheuvel-Leliefeld et al., 2015; Guidi et al., 2016). These limitations highlight the need for a new class of antidepressants and antipsychotics, particularly for patients with treatment-resistant disease.

There is ample evidence that inflammation plays a central role in the pathophysiology of depression and schizophrenia (Dantzer et al., 2008; Potvin et al., 2008; Girgis et al., 2014; Gold, 2015; Hashimoto, 2015; Steullet et al., 2016; Marques et al., 2018; Swardfager et al., 2018). In patients not undergoing antidepressant therapy, meta-analyses have reported that blood levels of proinflammatory cytokines, including tumor necrosis factor-α (TNF-α) and interleukin 6 (IL-6), are significantly higher than in healthy controls (Dowlati et al., 2010; Young et al., 2014; Haapakoski et al., 2015; Strawbridge et al., 2015). Moreover, studies of postmortem brain samples revealed increased proinflammatory cytokine gene expression in the prefrontal cortex (PFC) of individuals with a history of depression (Dean et al., 2010; Shelton et al., 2011). Studies using animal models of depression have reported lipopolysaccharide (LPS)-induced depression-like behavior and dendritic changes (Zhang et al., 2015, 2016). In studies investigating schizophrenia, the levels of proinflammatory cytokines, such as TNF-α, and IL-6, were significantly elevated in the serum and cerebrospinal fluid (Sasayama et al., 2013; Upthegrove et al., 2014; Schwieler et al., 2015; Dickerson et al., 2016). Postmortem studies and meta-analyses have demonstrated increased microglia density and activity in patients with schizophrenia (van Kesteren et al., 2017; Marques et al., 2018). Several studies have demonstrated that anti-inflammatory drugs exhibit antipsychotic activity in animal models of schizophrenia (Shirai et al., 2012, 2015). Collectively, these investigations demonstrate that inflammation is likely closely related to depression and schizophrenia, and that anti-inflammatory drugs could effectively improve the symptoms of depression and schizophrenia.

## Soluble Epoxide Hydrolase and the Arachidonic Pathway

**Figure 1:**
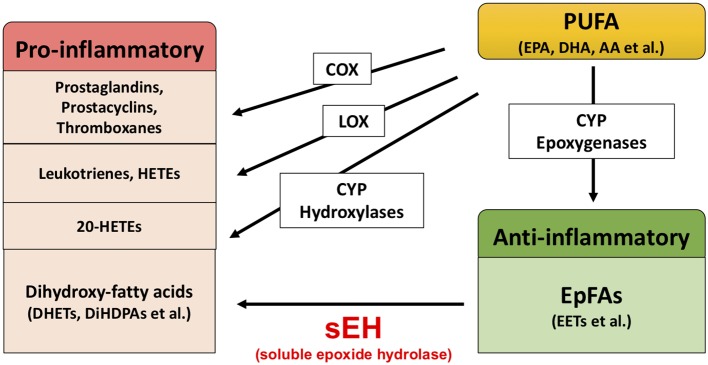
Brief overview of arachidonate cascade.

Extensive evidence suggests that biological substances in the arachidonate cascade, such as enzymes and eicosanoid metabolites, are involved in the etiology and pathology of inflammatory disease. Polyunsaturated fatty acids (PUFAs), such as arachidonic acid (AA), docosahexaenoic acid (DHA), and eicosapentaenoic acid (EPA), are metabolized by cyclooxygenases (COXs), lipoxygenases (LOXs), and cytochrome P450s (CYPs) (Figure 1; Imig and Hammock, 2009; Imig, 2012, 2018; Morisseau and Hammock, 2013). The COX and LOX pathways lead to the production of prostaglandins, leukotrienes, and hydroxyeicosatetraenoic acids (HETEs). These lipid mediators are involved in pro-inflammatory processes, while lipoxins synthesized from AA by LOX play an important role in the resolution of inflammation (Serhan and Savill, 2005; Serhan, 2017a,b). In contrast, the CYP pathway is involved in both the production of pro-inflammatory lipid mediators and anti-inflammatory lipid mediators. The CYP hydroxylases lead to 20-HETE (pro-inflammatory mediator), and CYP epoxygenases lead to epoxyeicosatrienoic acids (EETs) (anti-inflammatory mediator). Through catalyzing the epoxidation of PUFAs, such as AA, the epoxygenase CYP enzymes generate four regioisomeric EETs, including 5,6-EET, 8,9-EET, 11,12-EET, and 14,15-EET. However, the EETs are metabolized by soluble epoxide hydrolase (sEH) and converted into their corresponding diols (dihydroxyeicosatrienoic acids [DHETs]), and these molecules are considered to be less biologically active than their parent versions. Therefore, it is probable that sEH plays a role in the pathogenesis of several diseases caused by inflammation (Imig, 2005, 2012, 2016, 2018; Iliff et al., 2010; Morisseau and Hammock, 2013; Wagner et al., 2014, 2017; Zhang et al., 2014; Hashimoto, 2016).

## Role of sEH in Psychiatric Disorders

As discussed above, inflammation is associated with psychiatric disorders such as depression and schizophrenia, and sEH plays a role in the pathogenesis of inflammatory-related diseases. Therefore, it is possible that sEH contributes to the pathophysiology of these disorders. Recently, our study of postmortem brains revealed that the protein levels of sEH in the parietal cortex of patients with major depressive disorder, schizophrenia, and bipolar disorder were significantly elevated compared with those in the parietal cortex of healthy individuals (Ren et al., 2016). In studies involving mouse models, the brain, after inflammation or chronic social defeat stress, exhibited increased expression of sEH in the PFC and hippocampus. Meanwhile, after inflammation or social defeat stress, mice exhibited increased immobility time in tail suspension and forced swim tests, and decreased sucrose preference. These phenotypes were defined as depression-like behavior in mice (Duman and Monteggia, 2006; Dantzer et al., 2008). Studies involving behavioral tests in animal models have suggested that the sEH inhibitor 1-[1-propionylpiperidin-4-yl]-3-[4-(trifluoromethoxy) phenyl] urea (TPPU) had antidepressant effects because the inflammation and chronic social defeat stress-induced depression-like behavior was prevented by oral administration or chronic intake of TPPU (Ren et al., 2016). As such, TPPU could produce antidepressant effects in inflammation model of depression because standard antidepressants, such as selective serotonin reuptake inhibitors and serotonin norepinephrine reuptake inhibitors, do not demonstrate therapeutic effects in such models (Zhang et al., 2015). Moreover, the use of TPPU lowered serum TNF-α levels in LPS-treated mice but not control mice. Additionally, in these experiments, both TPPU and 14,15-EET potentiated nerve growth factor (NGF)-induced neuronal outgrowth in PC12 cells. Consistent with the pharmacological inhibition of sEH, despite experiencing chronic social defeat stress, sEH knockout mice did not exhibit depression-like behavior. It is likely that the deletion of the sEH gene conferred resilience to social defeat stress. Additionally, investigation of brain-derived neurotrophic factor-tropomyosin receptor kinase B (BDNF-TrkB) signaling protein expression in sEH knockout mice brains revealed that BDNF and p-TrkB-to-TrkB protein ratios were elevated in the PFC and hippocampus. Consistent with BDNF-TrkB signaling, the protein levels of glutamate receptor subunit (GluA1) and postsynaptic density protein (PSD-95), which are synaptogenesis biomarkers, were elevated in the PFC and hippocampus of sEH knockout mice. This could infer that deletion of sEH resulted in resilience to chronic social defeat stress *via* increased BDNF-TrkB signaling and synaptogenesis. Furthermore, data reported by other groups are in agreement with these results. Wu et al. reported that the sEH inhibitor TPPU decreased depression-like behavior in the novelty-suppressed feeding test, which is a test of stress-induced anxiety/depression (Wu et al., 2017). Moreover, treatment with TPPU elevated the expression of BDNF in the mouse hippocampus and PC12 cells, and the antidepressant effect of TPPU was blocked by a BDNF-TrkB signal pathway antagonist (Wu et al., 2017, 2019). This evidence suggests that BDNF is necessary for the antidepressant effects of TPPU. Collectively, these findings highlight a key function of sEH in the etiology and pathology of depression, and for its inhibitors as potential therapeutic or prophylactic drugs for depression (Ren et al., 2016; Hashimoto, 2016).

As mentioned above, patients with schizophrenia exhibit higher sEH protein levels in the parietal cortex than controls. Meanwhile, another study investigating alterations of eicosanoids in the serum of patients with schizophrenia reported that 11,12-DHETs and 14,15-DHETs were increased in patients compared with controls and were decreased post-treatment (Wang et al., 2018). This evidence suggested that EET and its metabolic enzyme sEH may play a role in schizophrenia, and studies using animal models have provided strong supportive data. Ma et al. (2013) investigated the effects of AS2586114, a potent sEH inhibitor, in an animal model of schizophrenia. In a phencyclidine (PCP)-induced model of schizophrenia, a single oral administration of AS2586114 attenuated PCP-induced hyperlocomotion in a dose-dependent manner. Furthermore, AS2586114 also improved PCP-induced prepulse inhibition deficits in a dose-dependent manner. In addition, AS2586114 exhibited a similar effect to the atypical antipsychotic drug clozapine in PCP-induced behavioral abnormalities (Ma et al., 2013). These studies suggest the therapeutic potential of sEH inhibitors for schizophrenia. However, the precise mechanism by which sEH inhibitors diminish PCP-induced acute behavioral effects in mice remains unclear. Nevertheless, some studies have provided valuable clues. Ribeiro et al. reported that omega-3 PUFAs (n3 PUFAs), but not clozapine, prevented polyinosinic:polycytidylic acid (poly I:C)-induced deficits in BDNF (Ribeiro et al., 2019). Because decreased BDNF-TrkB signaling has been suggested to play a role in the pathophysiology of schizophrenia (Giovanoli et al., 2015; Han et al., 2016), and sEH inhibitors increase the level of BDNF, it appears that sEH may play a role in schizophrenia *via* regulation of the BDNF-TrkB signaling pathway. There is ample evidence suggesting that oxidative stress also plays an important role in the pathophysiology of schizophrenia, and antioxidant agents have demonstrated antipsychotic effects in animal models of schizophrenia (Matsuzawa and Hashimoto, 2011; Reddy and Reddy, 2011; Yao and Keshavan, 2011; Shirai et al., 2012, 2015). Moreover, abnormalities in striatal dopamine levels are a hallmark of schizophrenia pathophysiology (Brisch et al., 2014; Nakao et al., 2019). Recently, Ren et al. (2018) reported that inhibition of sEH protected against MPTP (1-methyl-4-phenyl-1,2,3,6-tetrahydropyridine)-induced endoplasmic reticulum (ER) stress and oxidative stress in the brain. The immunoreactivity of sEH is present almost exclusively in astrocytes throughout the brain (Marowsky et al., 2009); however, deletion of the sEH gene suppressed MPTP-induced activation of microglia in the mouse striatum (Ren et al., 2018). Additionally, inhibition of sEH attenuated MPTP-induced dopaminergic dysfunction in the striatum (Ren et al., 2018). Other studies have consistently found that deletion of the sEH gene and pharmacological inhibition of sEH blocked MPTP-induced heme-oxygenase (HO-1) elevation (a redox-regulated protein) and caspase 12 activation (a hallmark of ER stress) (Huang et al., 2018). These findings indicate that sEH inhibitors are effective in attenuating MPTP-induced dopaminergic neurotoxicity, oxidative stress, and ER stress. Given the evidence described, there is a possibility that sEH inhibitors exert their antipsychotic properties by increasing EETs, modulating the BDNF-TrkB signaling pathway, protecting against oxidative stress, and improving dopaminergic dysfunction in the brain.

## Discussion

In this minireview, we highlighted recent studies that have demonstrated the potential of sEH as a therapeutic target in psychiatric disorders. Crucial data from Ren et al. (2018) and Ma et al. (2013) demonstrate that protein levels of sEH in the brains of depressed and schizophrenic patients are higher than in controls. Additionally, sEH protein levels are elevated in the brains of mice with a depression-like phenotype. These data suggest that increased levels of sEH in the brain cause enhanced metabolism of anti-inflammatory PUFA epoxides, such as EETs, EDPs, and epoxyeicosatetraenoic acids (EEQs), eventually leading to depressive symptoms. Furthermore, a single administration of sEH inhibitor has been reported to prevent depression-like phenotypes in the inflammation and chronic social defeat stress models of depression. These important findings indicate that sEH inhibitors may have a rapid onset of antidepressive action, which is similar to the rapid-acting antidepressant ketamine, but without any observable side effects (Hashimoto, 2016). Moreover, a single administration of sEH inhibitor has also been reported to rescue PCP-induced behavioral abnormality in mice. These antipsychotic effects of sEH inhibitor are similar to those of the atypical antipsychotic drug clozapine. This is noteworthy because sEH inhibitors also may have a rapid onset of antipsychotic action without any observable side effects.

Several hypotheses have postulated that inflammation may play a causative role in depression and schizophrenia (Schiepers et al., 2005; Brown and Derkits, 2010; Brown and Meyer, 2018; Chen et al., 2019). However, how do inflammatory cytokines influence behavior? In the brain, inflammatory cytokines, such as TNF-α, IL-6, and IL-1β, are elevated in the brain during inflammation or chronic stress (Strawbridge et al., 2015; Menard et al., 2017). These cytokines have been implicated in the activation of indoleamine-2,3-dioxygenase (IDO) and tryptophan dioxygenase (TDO), and promote the metabolization of tryptophan into formylkynurenine, a precursor of kynurenine. Furthermore, the metabolites of kynurenine, such as kynurenic acid and quinolinic acid, are involved in modulation of N-methyl-D-aspartate receptor and α-amino-3-hydroxy-5-methyl-4-isoxazolepropionic acid receptor, and can also activate monoamine oxidase. Subsequently, these metabolites are implicated in dopamine synthesis, behavioral abnormalities, and regulation of neurotrophic and metabolic signaling through BDNF and mTOR (Schiepers et al., 2005; Calcia et al., 2016; Ghasemi et al., 2017; Lima Giacobbo et al., 2018; Price et al., 2018; Chen et al., 2019).

**Figure 2:**
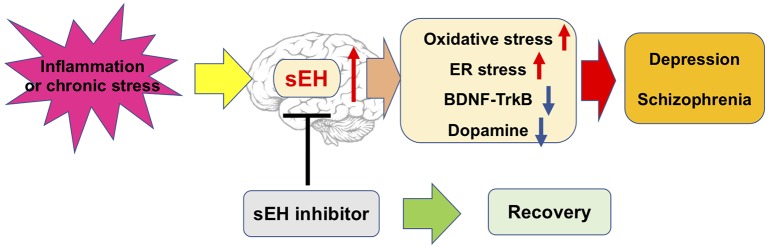
Possible mechanisms of the role of sEH in depression and schizophrenia.

Collectively, these studies described a potential mechanism for sEH in the pathophysiology of depression and schizophrenia. This evidence suggests that sEH inhibitors exert an antidepressant and antipsychotic effect, and may attenuate the appearance of oxidative and ER stress, dysregulation of neurotrophic and dysfunction of dopaminergic neurons in the brain (Figure 2). Nevertheless, the precise mechanism of action of sEH inhibitors remains largely unknown, thus warranting more in-depth studies in the future.

## Author Contributions

QR wrote the manuscript and prepared the figures.

### Conflict of Interest Statement

The author declares that the research was conducted in the absence of any commercial or financial relationships that could be construed as a potential conflict of interest.
